# Mortality in COPD patients discharged from hospital: the role of treatment and co-morbidity

**DOI:** 10.1186/1465-9921-7-109

**Published:** 2006-08-16

**Authors:** Gunnar Gudmundsson, Thorarinn Gislason, Eva Lindberg, Runa Hallin, Charlotte Suppli Ulrik, Eva Brøndum, Markku M Nieminen, Tiina Aine, Per Bakke, Christer Janson

**Affiliations:** 1 Department of Respiratory Medicine, Allergy and Sleep, Landspitali-University Hospital, Reykjavik, Iceland; 2 Department of Medical Sciences: Respiratory Medicine and Allergology, Uppsala University, Akademiska Sjukhuset, Uppsala, Sweden; 3 Department of Respiratory Diseases, Hvidovre Hospital, University of Copenhagen, Copenhagen, Denmark; 4 Department of Respiratory Medicine, Tampere University Hospital, Tampere, Finland; 5 Haukeland University Hospital, Bergen, Norway

## Abstract

**Background:**

The aim of this study was to analyse mortality and associated risk factors, with special emphasis on health status, medications and co-morbidity, in patients with chronic obstructive pulmonary disease (COPD) that had been hospitalized for acute exacerbation.

**Methods:**

This prospective study included 416 patients from each of the five Nordic countries that were followed for 24 months. The St. George's Respiratory Questionnaire (SGRQ) was administered. Information on treatment and co-morbidity was obtained.

**Results:**

During the follow-up 122 (29.3%) of the 416 patients died. Patients with diabetes had an increased mortality rate [HR = 2.25 (1.28–3.95)]. Other risk factors were advanced age, low FEV_1 _and lower health status. Patients treated with inhaled corticosteroids and/or long-acting beta-2-agonists had a lower risk of death than patients using neither of these types of treatment.

**Conclusion:**

Mortality was high after COPD admission, with older age, decreased lung function, lower health status and diabetes the most important risk factors. Treatment with inhaled corticosteroids and long-acting bronchodilators may be associated with lower mortality in patients with COPD.

## Background

Chronic Obstructive Pulmonary Disease (COPD) is associated with intermittent exacerbations characterized by acute deterioration in the symptoms of chronic dyspnea, cough and sputum production. Worldwide, COPD is the only leading cause of death that still has a rising mortality rate. It has been estimated that by the year 2020 COPD will be the third leading cause of death in the world [1]. Hospitalizations because of acute exacerbations are an important part of the care of patients with COPD. Furthermore, they are associated with further impairment of health status [2] and high cost [3]. Studies on mortality after hospitalization for an acute exacerbation of COPD have shown a one-year mortality from 22% [4] to 43% [5] and a 2-year mortality of 36 [6] to 49% [5].

Several studies have been conducted in order to identify the risk factors of mortality in COPD and there is a concomitant increasing interest in modifying the risk factors in order to reduce mortality. Among risk factors that have been identified in previous studies are increasing age, a higher PCO_2_, long-term use of oral corticosteroids [4], reduced health status, marital status, depression, co-morbidity and prior hospital admission [6]. There are limited data available regarding the relationship of inhaled medications to mortality. A retrospective study by Soriano *et al*. showed that outpatients treated with a combination of inhaled corticosteroids and long-acting beta agonists or inhaled corticosteroids alone had a lower mortality rate than those that were not so treated [7].

The aim of this study was to analyse prospectively mortality in COPD patients after hospitalisation and associated risk factors, with special emphasis on health status, medications and co-morbidity.

## Methods

This prospective study of patients hospitalised with acute exacerbations of obstructive airway disease in five university hospitals in the Nordic countries has been described previously [8,9].

The departments included were: The Department of Respiratory Medicine and Allergology, Akademiska sjukhuset, Uppsala, Sweden; The Department of Thoracic Medicine, Haukeland University Hospital, Bergen, Norway; The Department of Respiratory Medicine, Tampere University Hospital, Tampere, Finland; The Department of Respiratory Medicine, Vifilstadir University Hospital, Gardabaer, Iceland; and The Department of Respiratory Medicine, Hvidovre Hospital, Copenhagen Denmark. An Internal Review Board in each centre or country approved the study.

Consecutive patients from each of the participating hospitals were included, provided that they had been admitted with acute exacerbations of COPD during 2000–2001. An acute exacerbation was defined as a change in condition in a COPD patient from baseline that was of such a magnitude that the patient needed an acute hospital admission. All patients fulfilled the criteria for COPD according to stage 1 or higher of the Global Initiative for Chronic Obstructive Pulmonary Disease [10]. All records were reviewed by the investigators to confirm the diagnosis and GOLD criteria were used to diagnose COPD. Patients thought to have asthma were excluded. Only patients who were admitted for more than 24 hours were included. All patients signed an informed consent before entering the study.

The following data were collected at discharge from the respective pulmonary departments. Information was collected in a similar fashion on standardized data sheets in all the departments. All data were entered at one centre.

1. Questionnaire that included information on smoking history, type of living, and family situation (alone or with others).

2. Spirometry, body weight and height. Predicted values for forced expiratory volume in one second (FEV1) and forced vital capacity (FVC) were calculated based on the European Coal and Steel Union reference values [11]. COPD severity was calculated according to the GOLD-criteria [10].

3. Health status (quality of life) was assessed using the disease-specific St George's Respiratory Questionnaire (SGRQ). It has three components: symptoms, activity and impact, in addition to total score [12]. Higher scores indicate worse health status.

4. From the patients' records information was collected on treatment at discharge, including long-term oxygen therapy. The patients were categorized in four treatment categories based on the utilization of inhaled corticosteroids (ICS) and long-acting beta-2-agonists (LABA): none, only LABA, only ICS and both LABA and ICS [7]. Assessment of co-morbidity was based on the diagnosis used by the treating physician. Diabetes mellitus was considered to be present if the patient was using medication for diabetes. Hypertension, ischemic heart disease or atrial fibrillation was considered to be present when diagnosed by attending physician.

5. Two years after discharge information regarding death and causes of death was obtained from the National Registries in each country. The primary (underlying) cause of death was divided into the following categories: Respiratory causes [acute COPD exacerbations (ICD 10 code J44.0 and J44.1), respiratory insufficiency (J96) and pneumonia (J12-J18)]; Cardio-vascular causes [myocardial infarction (I21), heart failure (I50), stroke (I61 and I63) and rupture of aortic aneurysms (I71)]; Malignancy [lung cancer (C34), leukaemia (C91), lymphoma (C85) and abdominal tumour (D37)] and Other [septic shock (R57), aspiration (J69) and ileus (K56)].

## Statistics

Analyses were carried out using Stata 8.0 (Stata Corporation, College Station, Texas). The chi-square test and the unpaired t-test were used when comparing patients that had died during the study period. The relationshipbetween survival time and patient characteristics was determinedwith Kaplan-Meier survival analysis and Cox regression. Multivariate analyses also were carried out with theCox model after adjustment for FEV_1_. The analysed independent variableswere chosen based on statistical significance in the bivariateanalyses and on clinical relevance. Age, FEV_1 _and health status were entered as continuous variables, while gender, smoking status, previous hospitalizations, co-morbidity and treatment were entered as categorical variables. The proportional hazard assumption was tested for all the independent variables in the models and no violation was detected (p > 0.1). The effect of the pharmacological treatment at discharge was primarily assessed by entering the four LABA and ICS therapy categories and long-term oxygen to the model above. Other therapies were thereafter entered one at a time to the model. In order to detect heterogeneity between the hospitals concerning determinants for mortality the Cox regression estimates (hazard ratio) were also calculated by hospital and then combined, using random effect meta-analysis. A p-value of < 0.05 was considered statistically significant.

## Results

**Figure 1:**
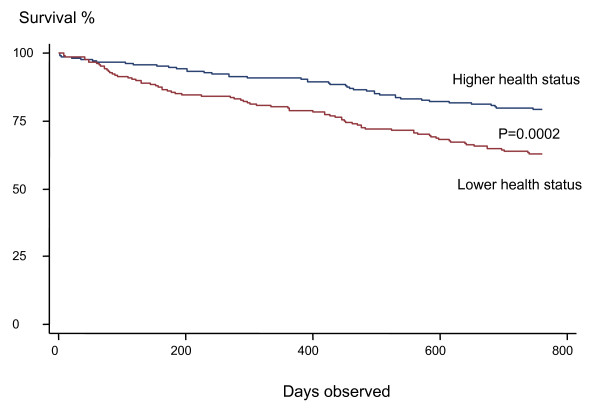
Kaplan-Meier survival curve in patients with higher (total SGRQ score ≤ 60) and lower health status (total SGRQ score > 60).

A total of 416 patients who were hospitalized for an acute COPD exacerbation between January 2000 and December 2001 were included in the study. During the two-year follow-up 122 (29.3%) of the 416 patients died. The primary cause of death was respiratory in 79 patients, cardiovascular in 21, malignancy in 7, other causes in 3 patients, whilst no information on causes of death was available for 12 patients. The patients that died were older, more often men, had worse lung function, and more often had a history of previous hospitalizations (Table 1). They also had a worse health status, both for total score and individual components. Patients with diabetes had a higher mortality rate (Figure 1).

**Table 1:** Differences between dead and surviving patients (mean ± SD or %).

	Alive (n = 294)	Dead (n = 122)	p-value
Age (years)	68.2 ± 10.9	72.1 ± 8.7	0.0005
Women	54.1	46.6	0.03
Current smokers	24.6	28.7	0.39
Pack years	35.7 ± 24.5	34.3 ± 19.8	0.59
Living alone	52.6	50.8	0.74
FEV_1 _(% pred)	40.6 ± 19.2	33.5 ± 14.4	0.0005
≥ 2 hospitalizations in previous 12 months	30.2	52.0	<0.0001
*Health status (SGRQ)*			
Symptoms	63 ± 20	69 ± 16	0.006
Activity	65 ± 22	72 ± 20	0.002
Impact	44 ± 19	51 ± 19	0.001
Total	56 ± 17	63 ± 16	0.0002
*Co-morbidity*			
Cardio-vascular disease	42.9	50.0	0.18
Diabetes	8.5	15.6	0.03
*COPD severity according to the GOLD classification *(12)			0.006
GOLD stage I-II	28	15	
GOLD stage III	31	29	
GOLD stage IV	41	57	

Mortality was related to older age, lower lung function, lower health status and diabetes, as shown in Table 2. Older age and diabetes were related to both respiratory and cardiovascular mortality. In addition respiratory mortality was related to lower lung function.

**Table 2:** Risk of dying in relation to primary cause of death. Cox regression, Hazard Risk ratio* and 95 % confidence interval.

	All deaths	Respiratory	Cardiovascular
Age (10 years)	1.49 (1.17–1.90)	1.45 (1.07–1.97)	2.62 (1.35–5.10)
Women	0.67 (0.44–1.03)	0.70 (0.41–1.21)	0.89 (0.32–2.48)
Current smoking	1.47 (0.91–2.38)	1.73 (0.97–3.10)	0.93 (0.18–4.86)
FEV_1 _(per 10% pred. change)	0.83 (0.71–0.96)	0.76 (0.62–0.92)	0.87 (0.61–1.25)
≥ 2 previous hospitalizations	1.22 (0.79–1.90)	1.33 (0.77–2.30)	1.35 (0.43–4.22)
*SGRQ score (4 units)*			
Symptoms**	1.04 (0.99–1.09)	1.03 (0.96–1.10)	1.06 (0.95–1.17)
Activity**	1.03 (0.98–1.08)	0.99 (0.93–1.04)	1.12 (0.98–1.28)
Impact**	1.06 (1.01–1.11)	1.07 (1.00–1.14)	1.09 (0.97–1.22)
Total score	1.07 (1.01–1.14)	1.05 (0.97–1.14)	1.14 (0.99–1.32)
*Co-morbidities*			
Diabetes	2.25 (1.28–3.95)	2.42 (1.18–4.96)	3.82 (1.15–12.8)
Cardio-vascular disease	1.43 (0.92–2.23)	1.52 (0.87–2.65)	1.96 (0.65–5.92)
*COPD severity according to the GOLD classification *(12)***			
GOLD stage I-II	1	1	1
GOLD stage III	1.18 (0.63–2.20)	1.09 (0.46–2.57)	0.69 (0.16–2.88)
GOLD stage IV	1.81 (1.02–3.24)	2.40 (1.13–5.12)	1.05 (0.27–4.06)

* adjusted for centre and the variables in the table

** entered separately, replacing SGRQ or HAD total score

*** entered separately, replacing FEV_1_

Table 3 compares medical treatment between the surviving and non-surviving groups. Treatment with inhaled corticosteroids and/or long acting beta-adrenergic inhalers was associated with decreased mortality compared to the group of seventy-four patients that were on neither of these types of therapy at discharge (Figure 3, Table 3). Nebulized bronchodilators and long-term oxygen use were also associated with increased mortality in the bivariate but not in the multivariate analyses. The group of patients that were not using inhaled corticosteroids or long-acting beta-adrenergics had a significantly lower usage of oral theophylline (17.6 vs. 29.5%, p = 0.03) than the groups of patients that were taking inhaled corticosteroids and/or long-acting beta-adrenergics, whereas no other differences were found concerning other types of maintenance therapy between these patient groups.

**Table 3:** Maintenance treatment at discharge (%) in relation to two-year mortality (ICS = inhaled corticosteroids, LABA = long-acting beta-2-agonists)

	Alive	Dead	p-value	Hazard risk ratio*
No ICS or LABA	13.4	30.4	<0.0001	1
ICS without LABA	22.1	19.1	0.51	0.30 (0.12–0.73)
LABA without ICS	14.5	7.8	0.07	0.45 (0.23–0.89)
Both ICS and LABA	50.5	42.6	0.15	0.47 (0.26–0.84)
Short acting beta-2-agonists MDI**	34.5	39.1	0.39	1.27 (0.78–2.08)
Ipratropium MDI**	33.8	38.4	0.38	1.07 (0.65–1.74)
Theophylline**	26.1	30.4	0.38	0.79 (0.48–1.30)
Nebulised beta-2-agonists and/or ipratropium**	27.2	49.1	0.0001	1.38 (0.83–2.28)
Long-term oxygen	20.8	30.3	0.03	1.07 (0.62–1.84)

* adjusted for age, sex, centre, smoking, FEV1, previous hospitalizations, SGRQ total score, co-morbidity and the variables in the tables

** entered separately into the model

No between-hospital heterogeneity was found in the association with the above risk factors and mortality when studied using meta-analysis (p for heterogeneity >0.1 in all analyses).

## Discussion

The present study is the first one to our knowledge to show that diabetes is a risk factor for mortality after hospitalization for an acute exacerbation of COPD. It is also the first prospective study to indicate that treatment with long-acting beta-agonists and inhaled corticosteroids is associated with lower mortality after hospitalization.

In the present study diabetes co-morbidity was related to a higher mortality rate. Studies have shown that hospitalized patients with diabetes have a high mortality rate. Previous studies have shown that patients with diabetes had a higher mortality rate after acute myocardial infarction [13] and cardiogenic shock [14] than did non-diabetic patients. Studies on COPD patients on co-morbidity and the relation to mortality have shown conflicting results. Almagro *et al*. [4] found a relation, whereas Groenewegen and co-workers [6] and Incalz and co-workers did not [16]. These studies all used the Charlson index for defining co-morbidity. Yohannes and co-workers did not find a relation with co-morbidity in elderly outpatients [16]. Connors *et al*. showed the influence of congestive heart failure and cor pulmonale on shortening survival time [5]. In our study cardiovascular co-morbidity was a risk factor only in those patients with lower health status (data not shown). Low health status had a stronger relation to cardiovascular than respiratory mortality, thus indicating that, in addition to COPD, cardiovascular co-morbidity adds to lower health status.

In the present study the use of inhaled corticosteroids and long-acting beta-adrenergic inhalers was associated with decreased mortality. A study by Soriano *et al*. on a total of 4665 outpatients from a general practice database showed three year survival to be higher in those 1045 patients who were regular users of inhaled corticosteroids alone or in combination with long-acting beta-adrenergic inhalers after adjustment for age, sex, smoking, co-morbidites and asthma [7]. His research was a retrospective study of outpatients with less severe COPD. Using a database of 22,620 patients Sin and Tu found that inhaled corticosteroids lowered the risk ratio for all causes of mortality by 29% in patients after hospitalization for COPD [19]. They also found that the use of oral corticosteroids was related to increased mortality, whereas bronchodilators had no effect on mortality [17]. It is of interest that our prospective study partly supported the results of these two retrospective studies as well as a more recent one [18]. In contrast to the previous studies we also found that the use of long-acting bronchodilators alone was related to a decrease in the mortality rate.

One advantage of the present study is that medication was assessed at discharge only, which avoids the problem with immortal time bias [19]. This has been reported as an important methodological issue in previous studies and subsequent studies have dealt with this point and not found survival benefits from inhaled corticosteroids [19-21]. A disadvantage is that we have no information on changes in therapy during the observation period. It should, however, be stated that both the present and the previous studies are observational and that a large randomized controlled study is needed to prove that COPD mortality can be reduced with inhaled corticosteroids and/or long-acting bronchodilators [22].

In the present study lower health status was related to higher mortality. This was true both for total score on the SGRQ and for the three subscales of activity, impact and symptoms. In the study by Almagro *et al*. the total score and the activity scale on the SGRQ showed a statistical difference [4]. A study by Fan and co-workers showed that those with the lowest quartile of physical function had a higher mortality during a one-year follow-up in an outpatient population [23]. A study by Oga of 150 male outpatients with COPD in Japan found that total score, activity and impact were related to mortality, whereas symptoms were not [24]. A study by Domingo-Salvany *et al*. on male outpatients reported that SGRQ and SF-36 total scores were independently associated with total mortality and respiratory mortality. [25]. Dyspnea was related to mortality in a study population that was followed after outpatient pulmonary rehabilitation [28]. In accordance with other studies we found that higher age [4-6,27] and worse lung function were related to an increased mortality rate [5,27]. There is an increasing interest in modifying risk factors in order to decrease hospital admissions and mortality. Several studies have shown that to be possible. Increasing physical activity has been shown to decrease both [29].

The mortality rates that we found following hospital admission for an exacerbation of COPD were slightly lower than in other reports. In a cohort of 1016 patients in the United States there was 43% mortality after one year and 49% after two years [5]. Groenewegen *et al*. found 23% mortality one year after hospitalization in 171 patients in the Netherlands [6]. A study from Spain on 124 men and 11 women showed a one-year mortality rate of 22% and a two-year mortality rate of 35.6% [4]. The lower mortality rate in our study may be explained by the fact that we studied different populations than in the other studies.

In the present study most of the 122 patients died from respiratory causes, a result that is similar to other studies [16,28]. A study of 215 COPD patients on LTOT found that the major causes of death were acute-on-chronic respiratory failure, heart failure, pulmonary infection, pulmonary embolism, cardiac arrythmia and lung cancer. It has, however, been suggested that relying on the information on death certificates underestimates COPD as the cause of death [30].

The present study included a fairly large number of patients, both males and females, and none were lost to follow-up regarding mortality data due to the excellent population registration in the Nordic countries. Causes of death are coded in a similar fashion in all the Nordic countries. The study has been carried out in several countries and represents a broad population of patients. However, there were also some weaknesses to our approach: The multicentre approach that can cause different database entries. Causes of death were based on death certificates that may not have been accurate and we did not get information on causes of death for all the patients that were included. For example, it has been shown that multidimensional grading systems are better than FEV1 to predict the risk of death [31]. There were also several things that are thought to be important in patients with COPD that there was no information on in the current study: For instance, we had no information on body mass index, physical capability and dyspnea that can be part of such grading systems. This may lead to residual confounding. In evaluating the association between treatment and mortality it is important to keep in mind that this was an observational study and not a randomized clinical trial.

## Conclusion

The present study has demonstrated clearly that mortality in patients after hospitalization with acute exacerbation of COPD was high and that the risk factors for mortality were older age, lower lung function, lower health status and diabetes co-morbidity. Our study also indicated that regular treatment with inhaled corticosteroids and long-acting bronchodilators was associated with lower mortality in severe COPD. These results should be taken into account when making clinical decisions about patients who have been admitted to hospital with acute exacerbations. Special emphasis should be put on the care of hospitalized patients that have both COPD and diabetes.

## Competing interests

The author(s) declare that they have no competing interests.

## Authors' contributions

GG participated in the design of the study and drafted the manuscript. TG participated in the design of the study and helped to draft the manuscript. EL participated in the design of the study and helped to analyse the data. RH helped to analyse the data. CSU participated in the design of the study, helped with interpretation of the data and helped to draft the manuscript. EB collected data for the study. MMN participated in the design of the study and interpretation of the data. TA collected data for the study. PB participated in the design of the study, performed statistical analyses and helped to draft the manuscript. CJ participated in the design of the study, performed statistical analyses and helped to draft the manuscript. All authors read and approved the final manuscript.

**Figure 2:**
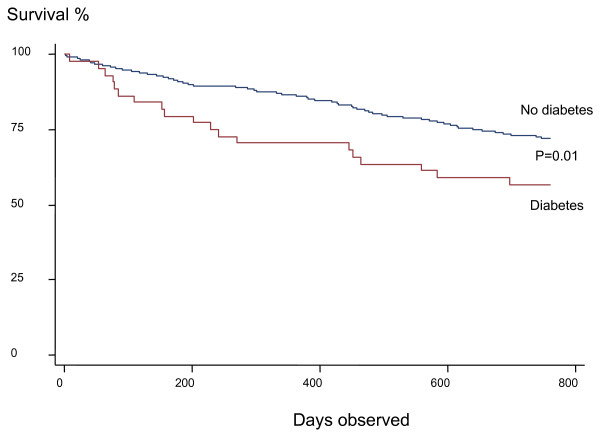
Kaplan-Meier survival curve in patients with and without diabetes.

**Figure 3:**
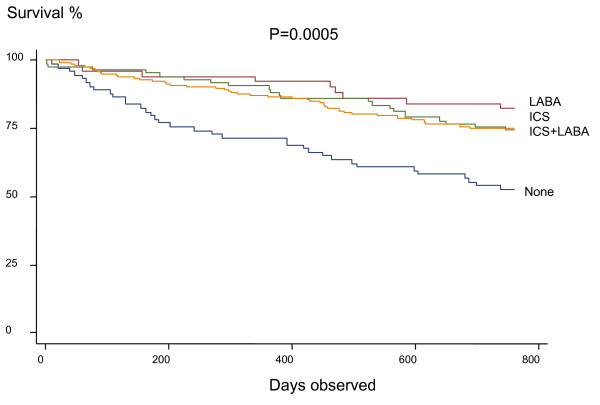
Kaplan-Meier survival curve in patients in relation to use of inhaled corticosteroids (ICS) and long-acting beta-2-agonists (LABA).

## References

[B1] Hurd S (2000). The impact of COPD on lung health worldwide: Epidemiology and incidence. Chest.

[B2] Seemungal TAR, Donaldson GC, Paul EA, Bestall JC, Jeffries DJ, Wedzicha JA (1998). Effects of exacerbation on quality of life in patients with chronic obstructive pulmonary disease. Am J Respir Crit Care Med.

[B3] Anderson F, Borg S, Jansson SA, Jonsson AC, Ericsson A, Prutz C, Ronmark E, Lundback B (2002). The costs of exacerbations in chronic obstructive pulmonary disease. Resp Med.

[B4] Almagro P, Calbo E, Ochoa de Echaguen A, Barreiro B, Quintana S, Heredia JL, Garau J (2002). Mortality after hospitalization for COPD. Chest.

[B5] Connors AF, Dawson NV, Thomas C, Connors AF, Harrell FE, Desbiens N, Fulkerson WJ, Kussin P, Bellamy P, Goldman L, Knaus WA (1996). Outcomes following acute exacerbation of severe chronic obstructive disease. Am J Respir Crit Care Med.

[B6] Groenewegen KH, AM Schols, Wouters EFM (2003). Mortality and mortality-related factors after hospitalization for acute exacerbation of COPD. Chest.

[B7] Soriano JB, Vestbo J, Pride NB, Soriano JB, Kiri V, Maden C, Maier WC (2002). Survival in COPD patients after regular use of fluticasone propionate and salmeterol in general practice. Eur Resp J.

[B8] Gudmundsson G, Gislason T, Janson C, Lindberg E, Hallin R, Ulrik CS, Brondum E, Nieminen MM, Aine T, Bakke P (2006). Depression, anxiety and health status after hospitalisation for COPD: A multicentre study in the Nordic countries. Respir Med.

[B9] Gudmundsson G, Gislason T, Janson C, Lindberg E, Hallin R, Ulrik CS, Brondum E, Nieminen MM, Aine T, Bakke P (2005). Risk factors for rehospitalization in COPD : Health status, anxiety and depression. Eur Resp J.

[B10] Global initiative For Chronic Obstructive Lung Disease (2005). Global strategy for the diagnosis, management, and prevention of Chronic Obstructive Pulmonary Disease. http://www.goldcopd.org.

[B11] European Community for Coal and Steel (1983). Standardisation of lung function tests. Clin Respir Phys.

[B12] Jones PW, Quirk FH, Baveystock CM, Littlejohns P (1992). A self-complete measure of health status for chronic airflow limitation: The St. George's Respiratory Questionnaire. Am Rev Respir Dis.

[B13] Jonas M, Reicher-Reiss H, Boyko V, Behar S, Grossman E (2003). Hospital and 1-year outcome after acute myocardial infarction in patients with diabetes and hypertension. J Hum Hypert.

[B14] Tedesco JV, Wright RS, Williams BA, Tedesco JV, Kopecky SL, Dvorak DL, Reeder GS, Miller WL, Mayo Coronary Care Unit Group (2003). Effects of diabetes on the mortality risk of cardiogenic shock in a community-based population. Mayo Clin Proceedings.

[B15] Incalzi RA, Fuso L, DeRosa M (1997). Co-morbidity contributes to predict mortality of patients with chronic obstructive pulmonary disease. Eur Resp J.

[B16] Yohannes AM, Baldwin RC, Connolly M (2002). Mortality predictors in disabling chronic obstructive pulmonary disease in old age. Age Ageing.

[B17] Sin DD, Tu JV (2001). Inhaled corticosteroids and the risk of mortality and readmission in elderly patients with chronic obstructive pulmonary disease. Am J Respir Crit Care Med.

[B18] Kiri VA, Bettoncelli G, Testi R, Viegi G (2005). Inhaled corticosteroids are more effective in COPD patients when used with LABA than with SABA. Resp Med.

[B19] Suissa S (2003). Effectiveness of inhaled corticosteroids in chronic obstructive pulmonary disease. Am J Respir Crit Care Med.

[B20] Suissa S (2004). Inhaled steroids and mortality in COPD: bias from unaccounted immortal time. Eur Resp J.

[B21] Fan VS, Bryson CL, Curtis RJ, Fihn SD, Bridevaux PO, McDonell MB, Au DH (2003). Inhaled corticosteroids in chronic obstructive pulmonary disease and risk of death and hospitalization: time-dependent analysis. Am J Resp Crit Care Med.

[B22] TORCH study group (2004). The TORCH (TOwards a Revolution in Copd Health) survival study protocol. Eur Resp J.

[B23] Fan VS, Curtis JR, Tu SP, McDonell MB, Fihn SD, Ambulatory Care Quality Improvement Project Investigators (2002). Using quality of life to predict hospitalization and mortality in patients with obstructive lung diseases. Chest.

[B24] Oga T, Nishimura K, Tsukino M, Sato S, Hajiro T (2003). Analysis of the factors related to mortality in chronic obstructive pulmonary disease: role of exercise capacity and health status. Am J Resp Crit Care Med.

[B25] Domingo-Salvany A, Lamarca R, Ferrer M, Garcia-Aymerich J, Alonso J, Felez M, Khalaf A, Marrades RM, Monso E, Serra-Batlles J, Anto JM (2002). Health related quality of life and mortality in male patients with chronic obstructive pulmonary disease. Am J Resp Crit Care Med.

[B26] Gerardi DA, Lovett L, Benoit-Connors ML, Reardon JZ, ZuWallack RL (1996). Variables related to increased mortality following out-patient pulmonary rehabilitation. Eur Resp J.

[B27] Anthonisen NR, Wright EC, Hodgkin JE (1986). Prognosis in chronic obstructive pulmonary disease. Am Rev Respir Dis.

[B28] Zielinski J, McNee W, Wedzicha JA, Ambrosino N, Braghiroli A, Dolensky J, Howard P, Gorzelak K, Lahdensuo A, Strom K, Tobiasz M, Weitzenblum E (1997). Causes of death in patients with COPD and chronic respiratory failure. Mon Arch Chest Dis.

[B29] Garcia-Aymerich J, Lange P, Benet M, Schnohr P, Anto JM (2006). Regular physical activity reduces hospital admission and mortality in chronic obstructive pulmonary disease: a population-based cohort study. Thorax.

[B30] Hansell AL, Walk JA, Soriano JB (2003). What do chronic obstructive pulmonary disease patients die from. Eur Resp J.

[B31] Celli BR, Cote CG, Marin JM, Casanova C, Montes de Oca M, Menez RA, Plata VP, Cabral HJ (2004). The body-mass index, airflow obstruction, dyspnea, and exercise capacity index in chronic obstructive pulmonary disease. N Engl J Med.

